# The Transcriptional Heat Shock Response of *Salmonella* Typhimurium Shows Hysteresis and Heated Cells Show Increased Resistance to Heat and Acid Stress

**DOI:** 10.1371/journal.pone.0051196

**Published:** 2012-12-07

**Authors:** Carmen Pin, Trine Hansen, Marina Muñoz-Cuevas, Rob de Jonge, Jesper T. Rosenkrantz, Charlotta Löfström, Henk Aarts, John E. Olsen

**Affiliations:** 1 Institute of Food Research, Norwich, United Kingdom; 2 National Food Institute, Technical University of Denmark, Søborg, Denmark; 3 National Institute for Public Health and the Environment (RIVM), Bilthoven, The Netherlands; 4 Department of Veterinary Disease Biology, University of Copenhagen, Copenhagen, Denmark; Instutite of Agrochemistry and Food Technology, Spain

## Abstract

We investigated if the transcriptional response of *Salmonella* Typhimurium to temperature and acid variations was hysteretic, i.e. whether the transcriptional regulation caused by environmental stimuli showed memory and remained after the stimuli ceased. The transcriptional activity of non-replicating stationary phase cells of *S.* Typhimurium caused by the exposure to 45°C and to pH 5 for 30 min was monitored by microarray hybridizations at the end of the treatment period as well as immediately and 30 minutes after conditions were set back to their initial values, 25°C and pH 7. One hundred and two out of 120 up-regulated genes during the heat shock remained up-regulated 30 minutes after the temperature was set back to 25°C, while only 86 out of 293 down regulated genes remained down regulated 30 minutes after the heat shock ceased. Thus, the majority of the induced genes exhibited hysteresis, i.e., they remained up-regulated after the environmental stress ceased. At 25°C the transcriptional regulation of genes encoding for heat shock proteins was determined by the previous environment. Gene networks constructed with up-regulated genes were significantly more modular than those of down-regulated genes, implying that down-regulation was significantly less synchronized than up-regulation. The hysteretic transcriptional response to heat shock was accompanied by higher resistance to inactivation at 50°C as well as cross-resistance to inactivation at pH 3; however, growth rates and lag times at 43°C and at pH 4.5 were not affected. The exposure to pH 5 only caused up-regulation of 12 genes and this response was neither hysteretic nor accompanied of increased resistance to inactivation conditions. Cellular memory at the transcriptional level may represent a mechanism of adaptation to the environment and a deterministic source of variability in gene regulation.

## Introduction

Natural environments are spatially and temporally complex. Bacteria interact with the environment responding to changes and changing the environment in return. Many studies on bacterial responses to environmental conditions focus on the quantitative analysis of growth, survival or inactivation of the population [1]. The investigation of the molecular response to the environmental conditions pursues a better understanding of the system bacteria-environment.

Hysteresis refers to a process by which a bistable system exhibits memory. Such systems switch between two distinct stable steady states, and switching from one state to the other happens when a stimulus exceeds a threshold. Once switched, the system remains at that steady state until the stimulus decreases to a level below the original switching level. In between these two switching stimulus levels the state of the system depends on the previous history [2]. Hysteresis has for example been described in the expression of components of the lactose utilization network of *Escherichia coli* [3]. In the absence of glucose, the *lac* operon is uninduced at low concentrations (<3 mM) of the inducer thio-methylgalactoside (TMG), and fully induced at high TMG concentrations (>30 mM). Between these switching thresholds, the response of the system is hysteretic: TMG levels must exceed 30 mM to turn on initially uninduced cells but must drop below 3 mM to turn off initially induced cells [3]. Thus, the pattern of lactose consumption adopted by bacteria is environmentally controlled and the key determinant is the direction of change of the environmental inducer [4].

The aim of the current study was to characterize the transcriptional responses of *Salmonella enterica* serotype Typhimurium (*S.* Typhimurium) to heat treatment and pH changes, specifically if these responses were hysteretic. We quantified the transcriptional response of stationary non-proliferating cells of *S.* Typhimurium to an increase of temperature from 25 to 45°C and a decrease of pH from 7 to 5. The conditions were maintained for 30 minutes before resetting the original values, and samples were taken at the end of the environmental stress, immediately after resetting the initial conditions and again 30 minutes later. Network science was applied to identify the metabolic pathways and functional categories in which the differentially transcribed genes were involved and confirmed the hysteretic behaviour of the transcriptional activity. In addition, the resistance to inactivating conditions of 50°C and pH 3 and the adaptation response to extreme growth environments at 43°C and pH 4.5 were monitored during the exposure to the stressing condition and immediately and 30 minutes after resetting the initial conditions.

## Results

### Acidification Affected Transcription of Few Genes, While a Large Number of Genes were Affected by Heat Shock

**Figure 1:**
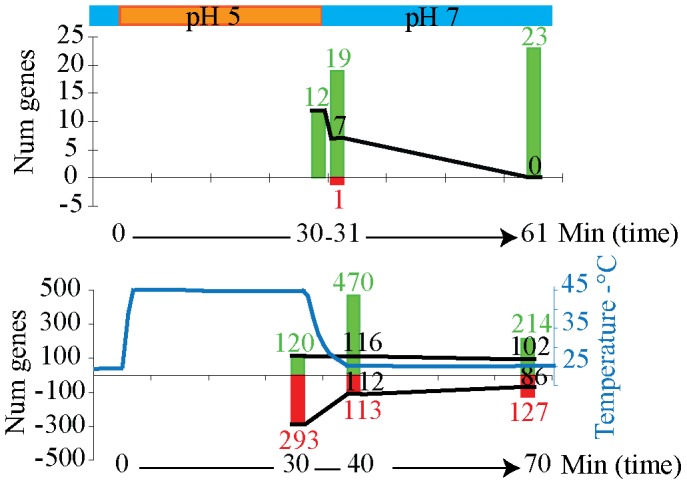
Number of genes up- (green) and down- (red) regulated in *S*. Typhimurium under acid and heat stressing conditions and immediately and 30 min after removing the stressing conditions. Few genes were affected by acid stress (A) while transcription of a large number of genes was altered under heat stress (B). Majority of up-regulated genes under heat stress remained up-regulated 30 minutes after stress condition ceased. Black numbers and solid lines show up- and down- regulated genes maintained throughout the experiment.

Only 12 genes were detected as up-regulated in a culture maintained for 30 minutes at pH 5 when compared with a control culture maintained at pH 7. Immediately after re-establishing the original pH value of 7 in the acidified culture, 19 genes were up-regulated and 1 gene was down-regulated. Thirty minutes after re-establishing the original conditions, 23 genes were up-regulated in the culture previously exposed to acidic conditions (Fig. 1). In contrast with these results, the increase of temperature from 25 to 45°C affected transcription of a much larger number of genes. The transcription of 293 genes was repressed while 120 genes were induced in the culture maintained at 45°C for 30 minutes when compared with control cultures at 25°C. Immediately after the heat shock ceased, the number of up-regulated genes increased considerably to 470 while the number of down-regulated genes decreased to 113. Thirty minutes after the heat condition was removed, there were still a large number of induced genes, 214, and a smaller number of down-regulated genes, 127, in the culture previously exposed to high temperature (Fig. 1). Thus, during the heat shock, most of affected genes showed repressed transcription while when the temperature was reset to the initial value, transcription was mostly induced with respect to control cultures.

The few up-regulated genes during the acid shock were associated with general metabolic and cellular functions (Fig. S1). In the acid stress experiment, the transcription of three genes associated to adaptation processes to atypical conditions *ibpB*, *pspB* and *pspC* was induced but only 30 minutes after the acid shock ceased. The induction of the phage shock protein operon, *pspABCE*, has been observed in response to a variety of stressful conditions [5], while the small heat-shock protein, IbpB, has been reported to stabilize stress-denatured proteins in *E. coli* [6].

The heat shock in particular caused the induction of genes encoding for chaperones, including heat shock proteins, and plasmid genes and the repression of genes involved in pathogenesis, energy production and motility. Some general pathways and functions were detected as both induced and repressed during all sampling times throughout the heat shock experiment (Fig. S2). However the majority of specific cellular functions or pathways included in these general categories had a consistent response being either up- or down-regulated throughout the experiment (Fig. S3).

### Most of Induced Genes in Response to Heat Stress Exhibited Hysteresis and Remained Induced after Resetting the Initial Conditions

From the 120 genes up-regulated in cultures exposed to 45°C for 30 minutes, 102 genes were still up-regulated 30 minutes after resetting the temperature to 25°C with respect to control cultures (Fig. 1). Conversely, none of the 12 genes up-regulated during the acidic shock was detected 30 minutes after the acidic condition was removed (Fig. 1).

**Figure 2:**
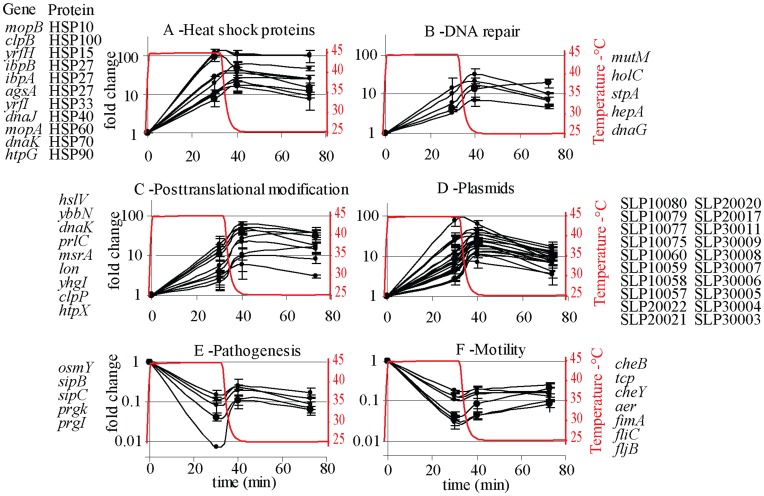
Fold change of transcript levels of genes during heat stress and immediately and 30 minutes after temperature was set back at 25°C that exhibited hysteresis and encoded for heat shock proteins (A), for DNA repair (B) for products involved in protein stabilization (C) or were encoded in plasmids (D). Down-regulated genes throughout the experiment were associated with non stress specific response such as pathogenesis (E) and motility (F). Genes represented in each plot showed a similar tendency; they are listed by the side of each plot but not differentially represented.

Thus, while the response to acid stress was very modest, heat shock caused a major alteration on gene transcription and this transcriptional response exhibited hysteresis. The majority of genes induced when cells were exposed at 45°C remained induced 30 minutes after the temperature was reset at 25°C. Among those genes, there were ten genes encoding for heat shock proteins as well as numerous genes encoding for other products involved in protein stabilization and DNA repair (Fig. 2). A significant number of genes encoded in the three plasmids of *S.* Typhimurium strain 4/74 also showed a hysteretic response (Fig. 2). Down-regulated genes throughout the experiment were associated with non-stress specific responses such as motility, pathogenesis and energy production (Fig. 2, Fig. S3).

### Down-regulation of Genes was Significantly Less Organized According to Cellular Functions or Metabolic Pathways than Transcriptional Induction during and after the Heat Shock

We constructed a genome scale bi-partite network for the genome and plasmids of *S*. Typhimurium SL1344 as previously described for *E. coli* K 12 [7]. The network was bipartite and thus edges connected two sets of nodes. Genes constituted one of these sets of nodes while the other set of nodes included metabolic pathways and cellular functions. Information was collected from public available resources and databases specified in the Material and Methods section. The bipartite sub-networks corresponding to genes up- and down-regulated during and after the heat shock were extracted from the genome scale network in order to study if network properties were affected by the environmental stresses.

**Figure 3:**
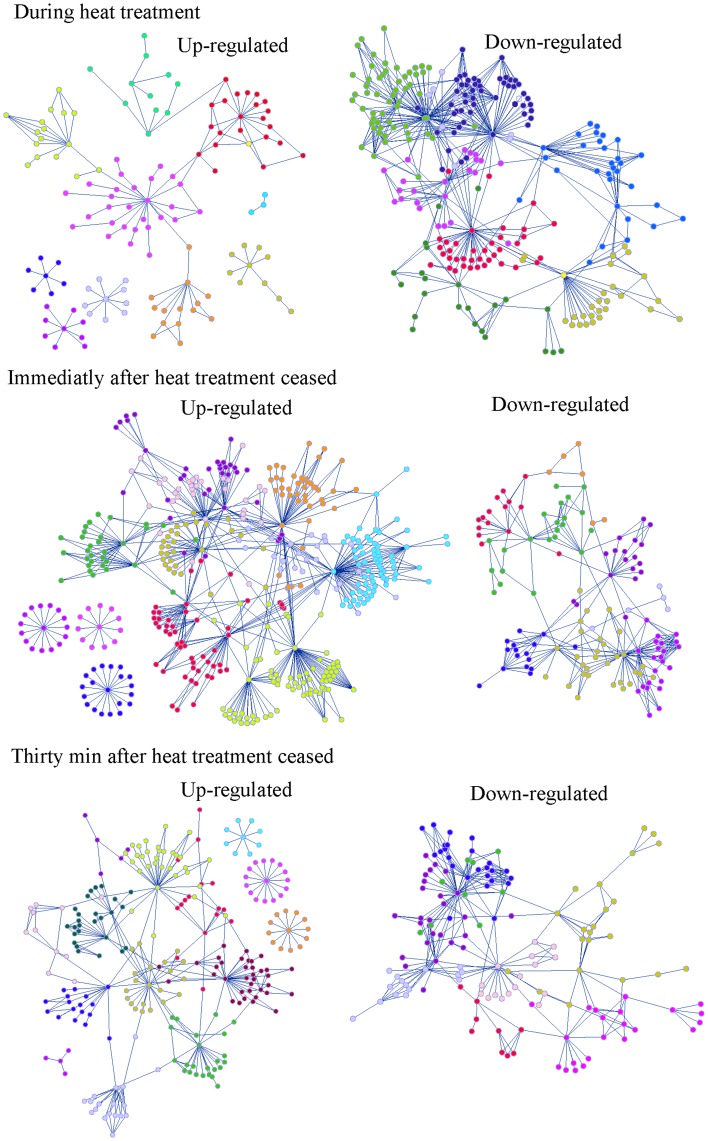
Network representation of genes up- and down-regulated during and after the heat shock. Modularity of networks of down-regulated genes was significantly lower than that of up-regulated networks. The induction of genes belonging to a metabolic pathway or cellular function was more synchronized than their down-regulation because of the hysteretic transcriptional response or persistence of the induction of the majority of genes induced by the heat shock once the temperature is set back to 25°C. Different colours represent different modules in each network.

The genome scale network was structured in modules or communities of nodes more connected to the nodes belonging to the same module than to other external nodes. To quantify this organization in communities the modularity value, Q [8], was calculated. The value of Q varies between 0 to a maximum value of 1. In practice it is found that a value above about 0.3 is a good indicator of significant community structure in a network [8]. The value of Q was 0.68 for our genome scale network. The modular or compartmentalized pattern of the genome network can be understood as a strategy to increase network stability, since this property retains the impact of perturbations within a single module and minimizes their effect on other metabolic pathways or cellular functions [9]. Modularity was estimated on the sub-networks from the genome scale network corresponding to genes differentially expressed during and after the heat treatment (Table 1). To assess the significance of the differences in the modularity of the sub-networks of the genes up- and down-regulated during and after the heat shock, we compared these results with the modularity measured in 10 random networks, with the same number of genes as the networks of up- and down-regulated genes but randomly selected from the genome scale network. Data in Table 1 shows that the value of the modularity coefficient for the networks of up-regulated genes during and after the heat treatment were between 0.75 and 0.8. These modularity values were slightly higher although not significantly different from those measured in the correspondent randomly generated networks. Fig. 3 shows the modular organization of the networks of genes differentially transcribed during the heat shock. Plasmid genes formed 3 disconnected modules that increased the modularity coefficient of the networks of induced genes (Fig. 3). Removing the modules formed by plasmid genes, the modularity coefficient of the networks of induced genes decreased slightly, taking values between 0.73 and 0.77 which are in the range of those of the random networks. Plasmid genes were not connected to other functions because the knowledge on their function is limited. Plasmid genes are known to be involved in intra-macrophage survival of *Salmonella*, antibiotic resistance and increased resistance to phage infection but their annotation is not public yet [10].

**Table 1:** Modularity quantification in sub-networks extracted from the *Salmonella* genome scale network containing those genes differentially expressed during and after the heat treatment and comparison with random extracted sub-networks with the same number of genes.

	Num genes & Num other nodes			Modularity coefficient (Q)^1^				
	Up-regulated	Down-regulated		Up-regulated	Random networks		Down-regulated	Random networks
End of heat treatment(45°C-30 min)	93 & 38	211 & 53		0.81	0.79±0.042^2^		0.64*	0.74±0.034
Immediately after heat treatment ceases	373 & 64	94 & 41		0.76	0.71±0.037		0.63*	0.82±0.040
Thirty minutes after heat treatment ceases	187 & 61	103 & 43		0.80	0.78±0.041		0.62*	0.81±0.044

1 Modularity coefficient from 0 to 1 (maximum modularity).

2 Mean value and standard deviation of the modularity coefficient of 10 random networks.

* Significant: Smaller than the mean value minus 3 times the standard deviation of random networks.

Therefore the modular structure observed in the networks of up-regulated genes during and after the heat shock was the same as observed in the random networks derived from the genome scale network reflecting its organization in metabolic pathways and cellular functions. However, modularity analysis of the networks of down-regulated genes during and after the heat shock revealed that the level of organization of repressed genes in functional modules was smaller than expected. Networks of repressed genes showed modularity coefficients in between 0.62 and 0.64 while randomly extracted networks had significantly greater Q values of 0.74–0.81 (Table 1).

### Hysteretic Transcriptional Response to Heat Shock is Accompanied by Increased Resistance to Inactivation Conditions of 50°C and pH 3

The stressing conditions, 45°C or pH 5 for 30 minutes, were selected by choosing extreme environmental conditions that did not cause inactivation while challenging *S*. Typhimurium. To study if these stressing treatments had an effect on the ability of *Salmonella* to grow in extreme conditions, we followed the kinetics of the stressed populations at 43°C and at pH 4.5. On the other hand, to investigate if the exposure to stress conditions affected the resistance of the population to inactivation conditions, the kinetics of the stressed populations were measured at 50°C and at pH 3.

**Figure 4:**
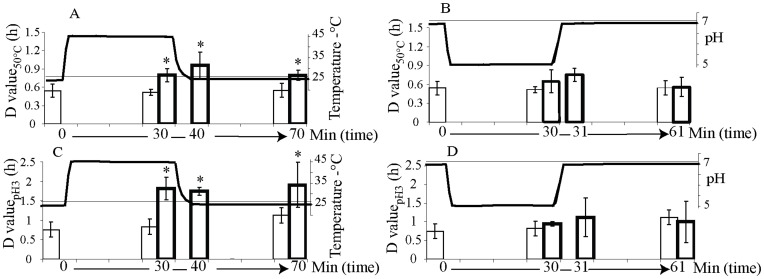
Increased resistance to heat and acid inactivation conditions in cultures of *S*. Typhimurium previously exposed to heat stressing conditions holds 30 min after resetting the initial conditions. Columns represent *D* values, time required for a decimal reduction of the population at the inactivating conditions, while lines represent the previous temperature or pH profile for each culture. Thick strokes are used for results at stressing profiles, 45°C or pH 5, and thin strokes for control experiments at 25°C and pH 7. D values at 50°C (A) and pH 3 (C) were significantly greater in cultures exposed to heat shock, 45°C for 30 min (thick strokes), and immediately and 30 min after the temperature was set back at 25°C than D values of control cultures at 25°C (thin strokes). However D values at 50°C (B) and pH 3 (D) of cultures previously exposed at pH 5 (thick strokes) were not significantly different from D values of control cultures at pH 7 (thin strokes). Stars indicate those cultures exposed to stressing conditions with D values significantly different from correspondent controls.

Cultures exposed at 45°C for 30 minutes exhibited an increased resistance to inactivation temperatures of 50°C as well as cross-resistance to inactivation by acidic conditions at pH 3 showing significantly greater D values than non-heated control cultures (Fig. 4). The D-values measured in cells exposed at 45°C for 30 minutes were not significantly different from those measured immediately and 30 minutes after the heat shock was removed (Fig. 4). Thus, the increased resistance to heat and acid inactivation conditions in cells exposed at 45°C for 30 minutes persisted for 30 minutes after the temperature was reset to 25°C. However, cultures exposed at pH 5 for 30 minutes did not show increased resistance to either heat or acid inactivation conditions when compared with control cultures kept at pH 7 (Fig. 4).

The growth parameters, duration of the lag phase and maximum specific growth rate, measured at either 43°C or at pH 4.5 were not affected by the previous exposure of the cells to 45°C or to pH 5 (Table 2).

**Table 2:** Duration of the lag phase and maximum specific growth rate at 43°C and pH 4.5 of *S.* Typhimurium previously exposed to heat and acid shock.

	43°C			pH 4.5	
Cell history	lag (h)		<$>\raster(80%)="rg1"<$> (h^−1^)	lag (h)	<$>\raster(80%)="rg2"<$> (h^−1^)
End of heat shock (45°C-30 min)	2.24±0.864*	0.911±0.136		20.5±0.636	0.444±0.0745
Immediately after heat shock and resetting temperature at 25°C	2.35±0.856	1.01±0.142		21.6±0.675	0.627±0.0804
Thirty minutes after heat shock and resetting temperature at 25°C	2.37±0.817	1.09±0.0378		22.1±0.169	0.687±0.0242
Control (25°C-pH 7-0 min)	2.56±0.878	1.12±0.152		22.1±0.0834	0.713±0.0605
Control (25°C-pH 7-30 min)	2.67±0.922	1.01±0.079		21.4±0.0476	0.57±0.0331
Control (25°C-pH 7-60 min)	1.9±0.00354	0.982±0.237		21.6±0.124	0.598±0.00135
End of acid shock (pH 5-30 min)	2.58±0.921	1.17±0.106		21.7±0.397	0.636±0.0472
Immediately after acid shock and resettingpH value at 7	2.57±0.889	1.09±0.183		21.6±0.122	0.591±0.0402
Thirty minutes after acid shock and resettingpH value at 7	2.4±0.992	0.951±0.309		21.8±0.389	0.617±0.0694

* mean±standard deviation of 3 independent replicated experiments.

## Discussion

The transcripts of the heat or acid shocked *S*. Typhimurium cells were compared with those of an untreated control kept at 25°C and pH 7 during the experimental course. We conducted growth curves to check that the populations used in the experiments were in early stationary phase after 16 hours at 25°C (data not shown), and thus mostly comprising non-proliferating cells although eventually some bacterial cells could divide or die. We targeted the use of non proliferating populations in order to avoid the interference of division cycle genes in the transcriptional response and the generation of new bacterial cells, which may not preserve the cellular memory of their ancestors. The bacterial concentration measured at early stationary phase was not affected during or after the application of the stressing conditions (Fig. S4).

We found a hysteretic transcriptional response to heat shock accompanied by an increased resistance to heat and acid inactivation conditions. The results supports previous observations that the resistance of *S.* Typhimurium to heating at 55°C was enhanced by exposing cells to a previous heat shock at 48°C and this thermotolerance was accompanied by increased synthesis of heat shock proteins. When cells were shifted from 48 to 37°C, thermotolerance was lost with a variable rate of decay within the first hour after the temperature shift while the synthesis of proteins persisted for longer time [11]. Thus, hysteretic behaviour is detectable at both transcription and translation and may be one of the reasons for the expression of apparently unneeded proteins that reduce growth rate of cells and is known as protein cost or burden [12]. We observed that the transcriptional response of *S.* Typhimurium to the acidification of the medium was not hysteretic and did not last after the acidic condition was removed. The decrease of pH from 7 to 5 did not seem to be a challenging stress condition for *S.* Typhimurium because it caused a very modest and unspecific transcriptional response and was not associated with either an increase of the resistance to inactivation conditions or with adaptation to extreme growth conditions. It seems that there was a lack of specific transcriptional response in *S.* Typhimurium exposed to pH 5. The few differentially transcribed genes detected at pH 5 may reflect the high variation intrinsic to gene transcription in bacteria. More than 60 genes have been reported to exhibit between 2–5 folds difference in the expression ratios when the strains being compared were grown in identical conditions but in different batches [13]. We think that one of the reasons for the different responses of *S* Typhimurium to the different environmental stresses may be that while a pH value of 5 does not challenge *Salmonella* spp., 45°C is a more stringent condition close to the limiting growth temperature. *Salmonella* spp. is able to grow at pH values below 4, while growth is not detected above 48°C. Moreover, culturing *Salmonella* to stationary phase in media containing glucose has been reported to induce acid tolerance response [14]. Cultures in stationary phase might have an enhanced transcription of genes involved in acid tolerance response, which could contribute to explain the lack of differences between the transcriptional response in control and in acid stressed populations.

A wide range of stabilities has been observed for individual mRNAs of *E. coli*, although approximately 50% of all mRNAs had half-lives shorter than 3 minutes and all of them shorter than 15 minutes [15]. Similar results have been observed for *Staphylococcus aureus* in stationary phase, although during exponential growth the percentage of mRNAs with a half-life shorter than 2.5 minutes increased to ca. 85% [16]. An increase of the turnover time of mRNAs has been reported under heat shock with the extended half-life of some transcripts being longer than 30 minutes [17], [18]. One of these studies reported the effects of heat shock not on few transcripts but on the global response on RNA half-lives. During heat shock approximately 60% of log-phase transcripts of *S. aureus* had a longer half-life than 5 minutes, but only 7.1% of them were stable for 30 minutes under heat stress [17]. The increase of the half-life of mRNAs in response to heat shock is not likely to be the explanation for the post heat shock detection of transcripts that we are reporting, and there are basic differences between those studies and our results. The extension of half-life is reported during the heat shock and it may be attributable to a failure in RNA degradation [18], while we are reporting that genes remained induced 30 min after the heat shock ceased. In addition, while during the heat treatment only 7% of genes were found to have a half life longer than 30 min [17], in our work we are reporting that 96% of the genes induced during the heat shock were detected immediately when the temperature was reset to 25°C and 85% of them were still induced 30 minutes later. Thus our results seem to be due to an hysteretic behaviour of transcription according to the cell pre-history rather than to the extension of the half-life of the transcripts. However, the switching levels for the induction/repression of heat shock genes cannot be determined from our data and it is unknown how this hysteretic response is affected by cell division, or by other environmental stimuli impacting on transcription.

The modularity of the structure of the genome scale network derives from the network construction itself and means that in general genes can be grouped in functional modules according to the cellular functions and/or metabolic pathways in which the proteins they encode are involved. Modularity was estimated on the sub-networks from the genome scale network corresponding to genes differentially expressed during and after the heat treatment. The obtained modularity values were different from the value exhibited by the genome network; however, this is expected in sub-networks of smaller dimension extracted from a primary larger network. Therefore, to assess the significance of the differences in the modularity of the sub-networks of the genes up and down-regulated during the heat shock, we compared their modularity values with those measured in 10 random networks. The random networks used for comparison had the same number of genes as the networks of up- or down-regulated genes; the genes were randomly selected from the genome scale network. The modularity values of the up-regulated network were not significantly different from those measured in the correspondent randomly generated networks. Therefore the modular structure observed in the networks of up-regulated genes during and after the heat shock was the was not significantly different from that observed in the random networks derived from the genome scale network, reflecting its organization in metabolic pathways and cellular functions. However, modularity analysis of the networks of down-regulated genes during and after the heat shock revealed that the level of organization of repressed genes in functional modules was smaller than expected.

Repressed genes showed a lower level of organization in metabolic pathways and functional categories than expected while induced genes maintained the level of modularity expected in sub-networks derived from the genome scale network. Gene induction, during and after the heat shock, exhibited hysteresis and was organized in metabolic pathways and functional categories. On the other hand, although a large number of genes (293) were repressed during the heat shock only one third of them remained repressed immediately and 30 minutes after the heat shock ceased and the networks of these repressed genes in metabolic and functional modules were significantly less organized in metabolic pathways and functional categories. Possibly gene repression is less synchronised than gene induction as a result of the hysteretic behaviour of induction. If genes remained induced once stimuli cease, gene repression which is the complementary event to induction has to be affected.

The hysteretic behaviour of gene transcription may mechanistically be explained by a dynamic switching threshold that changes according to the state of the gene. The concentration of inducer needed to initiate transcription of uninduced genes may be higher than that needed to maintain transcription of induced genes which may be coupled with metabolic reaction rates. Bistability can arise from substrate inhibition or product activation in metabolic pathways [19]. It is also possible that the switching threshold is not affected but the signal of the inducer is amplified by the induced gene in a positive feedback mechanism. In fact, all known bistable signalling systems contain a “positive” circuit such as the double-negative feedback in the artificial genetic circuit for *E. coli* [20], the positive feedback loop in the genetic network described for *Saccharomyces cerevisiae* [21] or in the transcriptional network of *Bacillus subtilis* leading to biofilm formation, sporulation and the generation of multiple distinct phenotypes within an homogeneous population [22]. However, further studies are needed to explain the exact mechanism behind the hysteretic responses we have observed in the current study.

Monitoring transcription at genome scale by microarray hybridization is usually associated with very high variability between replicates. In the experiments carried out in this work we detected 1931 genes significantly differentially regulated at least in one sample but only 856 (44%) were detected in replicated samples from independent cultures (data not shown). We estimated the number of false detected differentially transcribed genes due to experimental error based on hybridizations of identical samples. We detected only 60 genes with variable transcriptional results in 6 hybridizations of identical samples to slides with printed products for all genes in the genome (>4000). Thus, experimental error cannot be the cause of 1075 false detections. Recent studies have focussed on investigating stochastic fluctuations in gene expression [23], [24] describing intrinsic noise resulting from stochasticity in the biochemical reactions taking place at the gene level and extrinsic noise originated from fluctuations in other cellular components involved in gene expression. The extrinsic component of the noise is dominant with a major contribution to the variability of gene expression [24] and being propagated in the gene network to affect expression fluctuation of its downstream genes [25]. These studies highlight the importance of stochastic fluctuations on the variability of gene regulatory networks. In addition, our results suggest that the hysteretic response associated to the history of cells may also contribute to explain this large variability of gene regulation. We have observed that 102 genes associated with response to heat shock may be either induced or repressed at 25°C, depending on the previous culture conditions. Regulation of some genes may be unexpected as a response to current conditions but explicable and determined by past environments. In addition, due to the network structure of gene regulation, the variable expression associated with the hysteretic response can be expected to be amplified when affecting the regulation of downstream genes contributing substantially to the large variability of gene expression.

We demonstrated that the hysteretic transcriptional response to the exposure to 45°C for 30 minutes was accompanied with increased resistance to heat and cross-resistance to acid inactivation conditions even 30 minutes after the stress has ceased. Thus, hysteresis may explain other cross-protection mechanisms against environmental stresses such as the reported higher thermotolerance of acid-adapted *Salmonella* cells [26]. This phenomenon could also explain the dependence of the duration of the bacterial lag phase on the previous growth conditions [27], [28]. Some studies have reported that non-replicating bacteria also remember previous environmental conditions and this cell memory seems to be associated to exposure time. *E. coli* starved for long periods in stationary phase maintained anaerobic metabolism, typical of stationary phase, during the lag period when inoculated in fresh media while young stationary cells switched immediately to aerobic respiration [7]. In another study, *Listeria monocytogenes* was incubated at no-growth a_w_ values (0.90) for days and it was observed that the longer the pre-incubation period, the faster the initiation of the subsequent growth at also low but growth permitting a_w_ values [29]. The lack of an adaptation response to extreme growth conditions involving the lag phase and/or growth rate in our experiments was most likely due to the short duration, 30 minutes, of the previous exposure to the stressing conditions.

In food and animal feed chains, producers are responsible for the safety of their products. Thus tracing the origin of accidental or deliberate microbial contamination of feed and food is essential to establish corrective actions that prevent this contamination [30]. Hysteretic responses to environmental conditions and stresses associated with food production and process could be investigated to infer the time and point of contamination throughout the food chain. The detection of proteins and/or transcripts associated with past environments might represent a rapid inferring system for the reconstruction of the contamination scenario.

## Methods

### Bacterial Strain, Environmental Culture Conditions and Sample Preparation

*Salmonella enterica subsp. enterica* serovar Typhimurium strain 4/74 which is the parental strain of the *hisG* mutant SL1344 [31] was subcultured twice in tryptone soy broth (TSB, Oxoid, Basingstoke, UK) and incubated at 25°C for 24 hours. before being inoculated in 200 ml of TSB and incubated at 25°C for 16 hours to early stationary phase. This culture was divided in two parts; one untreated control culture kept at 25°C and pH 7 while the other part of the culture underwent stressing conditions either at pH 5 or at 45°C.

The temperature shift was carried out by moving the culture from a 25°C water bath to one at 45°C. Once the culture reached 45°C, it was left there for 30 minutes before being moved back to 25°C. The change of temperature with time was measured by a thermocouple applied to a replicate bottle of uninoculated medium. For the pH stress condition, the pH was lowered to pH 5 with hydrochloride acid and after 30 minutes changed back with sodium hydroxide to the original pH. Solutions to adjust the pH of the media were highly concentrated to avoid the dilution of the population and the possibility of growth initiation. Samples from the untreated control populations were processed at the same sampling times as the stressed cultures.

RNA samples for gene expression analysis and culture samples for growth and heating experiments were obtained before applying the stress, after 30 minutes with stress, immediately after the removal of stress and 30 minutes after the removal of stress. At each sampling time, 10 ml culture was harvest by adding 10 ml Ambion RNA*later* Tissue Collection solution (Life Technologies, Taastrup, Denmark) and placed at 4°C overnight and stored at −20°C until RNA extraction. Samples of all cultures were used straight away for growth and inactivation experiments.

Three independent biological replicates for both pH and temperature stress were run.

### Growth and Inactivation Experiments

One ml of each sample was inoculated in 100 ml of TSB to reach a concentration of ca. 10^7^ cfu/ml. For extreme growth conditions experiments, cultures were incubated either at 43°C or at pH 4.5. Growth was monitored by optical density (OD) at 600 nm. For inactivation experiments, cultures were incubated at either 50°C or pH 3 and bacterial concentration measured by plate counts on tryptone soy agar (TSA, Oxoid).

### Estimation of Growth and Inactivation Parameters and Statistical Analysis

The duration of the lag period and the maximum specific growth rate were estimated by fitting the model of Baranyi and Roberts [32] to the growth curves recorded as natural logarithm of OD measurements *vs* time while D-values, exposure time required for a decimal reduction of the population at constant inactivating temperatures, were estimated from the log linear inactivation curves of cfu/ml *vs* time.

A one way ANOVA model with one factor was fitted to the ranked growth and inactivation parameters. The factor of the model was the history of the cells previous to exposure to inactivating conditions or extreme growth environments and included 6 levels. Three levels referred to stressed cells and they were “end of stress” and “immediately” and “30 minutes” after initial conditions were reset. The other 3 levels refereed to control cultures maintained for “0”, “30” and “60” minutes at 25°C and pH 7.

Orthogonal contrasts were set up to investigate if growth an inactivation parameters of control cultures were significantly different from those of stressed cultures as well as the significance of the effect of the time spent at control conditions.

### DNA Microarray Hybridizations

Total RNA was purified from the RNA*later* solution using the RNeasy Mini Kit (Qiagen, Copenhagen, Denmark) according to the manufacturer’s instructions (“RNeasy Mini Protocol for Isolation of Total RNA from Bacteria”) with minor adjustments; for lysis, a 15 mg/ml lysozyme solution with proteinase K was used and on-column DNase digestion was performed. The quality of the RNA was checked using the NanoDrop (Fisher Scientific, Slangerup, Denmark). Labeled cDNA was synthesized from total RNA using the FairPlay III Microarray Labeling Kit (Agilent Technologies, Hoersholm, Denmark) according to manufacturer’s instructions without the Spike-in step. cDNA from the untreated cultures was labeled with Cy5 and cDNA from the stressed culture was labeled with Cy3. The labeled cDNA from the two cultures were mixed together and competitively hybridized on an 8×15 K Agilent microarray slide constructed for *Salmonella* Typhimurium strain SL1344 (deposited with GEO database ref. number: GPL15227) at 65°C for 17 hours, washed and scanned according to the “Two-Color Microarray-Based Prokaryote Analysis Protocol” (Agilent Technologies). The scanning was done using an Axon GenePix 4200A Microarray scanner (Axon, Foster City, CA) and the feature intensities were quantified using GenePix Pro 6.1 software (Axon, Foster City, CA).

### Microarray Data Analysis

Data normalization and analysis was carried out using the program ArrayLeaRNA (freely available at http://www.ifr.ac.uk/safety/ArrayLeaRNA/) which implements a Bayesian inference method based on the variability of the hybridization to internal controls probes in each array and operon predictions if available [33]. Genes detected as up- (down-) regulated at least in two of the three replicated samples were considered as differentially expressed for a given sampling time. In addition, genes up- (down-) regulated in only one replicate of a given sampling time were considered as differentially expressed if detected in at least two of the three replicates of the other two sampling times (Tables S1 and S2).

Microarray datasets have been deposited with GEO database (series accession number: GSE37636).

### Network Analysis

A bi-partite network was constructed for the genome and plasmids of *S*. Typhimurium SL1344 as previously described for *E. coli* K 12 [7]. Edges connected two sets of nodes. Genes constituted one of these sets of nodes. The genome composition was obtained from the Genome Project NCBI database [34]. The other set of nodes included metabolic pathways and cellular functions, according to the KEGG database [35], the CMR-TIGR database [36] and the COGs (Clusters of Orthologous Groups of proteins) functional categories obtained from the Genome Project NCBI database [34]. The number of nodes was 5153, from which 4717 were genes and the remaining 436 nodes represented metabolic pathways and cellular functions. There were 11626 edges between these two sets of nodes.

The genome scale network was used to extract the bipartite networks corresponding to genes up- and down-regulated during and after the heat shock. Only functional categories and metabolic pathways connected to a significant number of genes up or down-regulated were included in these networks. The significance was statistically evaluated assuming that number of genes up(down)-regulated belonging to a metabolic pathway or cell functional category follows the commonly assumed hypergeometric distribution as previously described [7].

For networks representation we used the program Cytoscape [37]. Networks modularity was estimated with the program implementing the fast modularity maximization algorithm [8].

## Supporting Information

Figure S1 **Metabolic pathways and cellular functions associated with those genes up-regulated during acid shock, immediately after acid shock ceased and 30 minutes after acid shock ceased.** Columns had positive values if functions were up-regulated and negative if down-regulated.
(TIF)

Figure S2 **Main metabolic pathways and general cellular roles with a significant (** ***p*** **<0.1) proportion of genes up- or down-regulated during heat stress (During), immediately (After) and 30 minutes (Later) after heat stress ceased.** Columns had positive values if functions were up-regulated and negative if down-regulated.
(TIF)

Figure S3 **Specific metabolic pathways and cellular sub-roles with a significant (** ***p*** **<0.1) proportion of genes up- or down-regulated during heat stress (During), immediately (After) and 30 minutes (Later) after heat stress ceased.** Columns had positive values if functions were up-regulated and negative if down-regulated.
(TIF)

Figure S4 **Bacterial concentration (closed symbols) during and after the exposure to stressing conditions**: A) heat shock at 45°C 30 minutes and B) acidification of the medium at pH 5 for 30 minutes. Concentrations were also measured immediately after the cease of the stressing conditions and 30 minutes after resetting the original conditions as well as in untreated control populations maintained at 25 °C and pH 7 during the experimental course (C). Significant variation of the bacterial concentration was not detected in any population under any condition.
(TIF)

Table S1 **Number of replicated samples in which genes were up- or down- regulated because of heat stress (45°C).**
(PDF)

Table S2 **Number of replicated samples in which genes were up- or down- regulated because of acid stress (pH 5) and comparison with results of expression under heat stress in Table S1.**
(PDF)
