# Supplementary figures and images for: The Transcriptional Heat Shock Response of Salmonella Typhimurium Shows Hysteresis and Heated Cells Show Increased Resistance to Heat and Acid Stress
Source: PLoS One. 2012 Dec 7;7(12):e51196. doi: 10.1371/journal.pone.0051196 (PMC3517412; doi:10.1371/journal.pone.0051196)

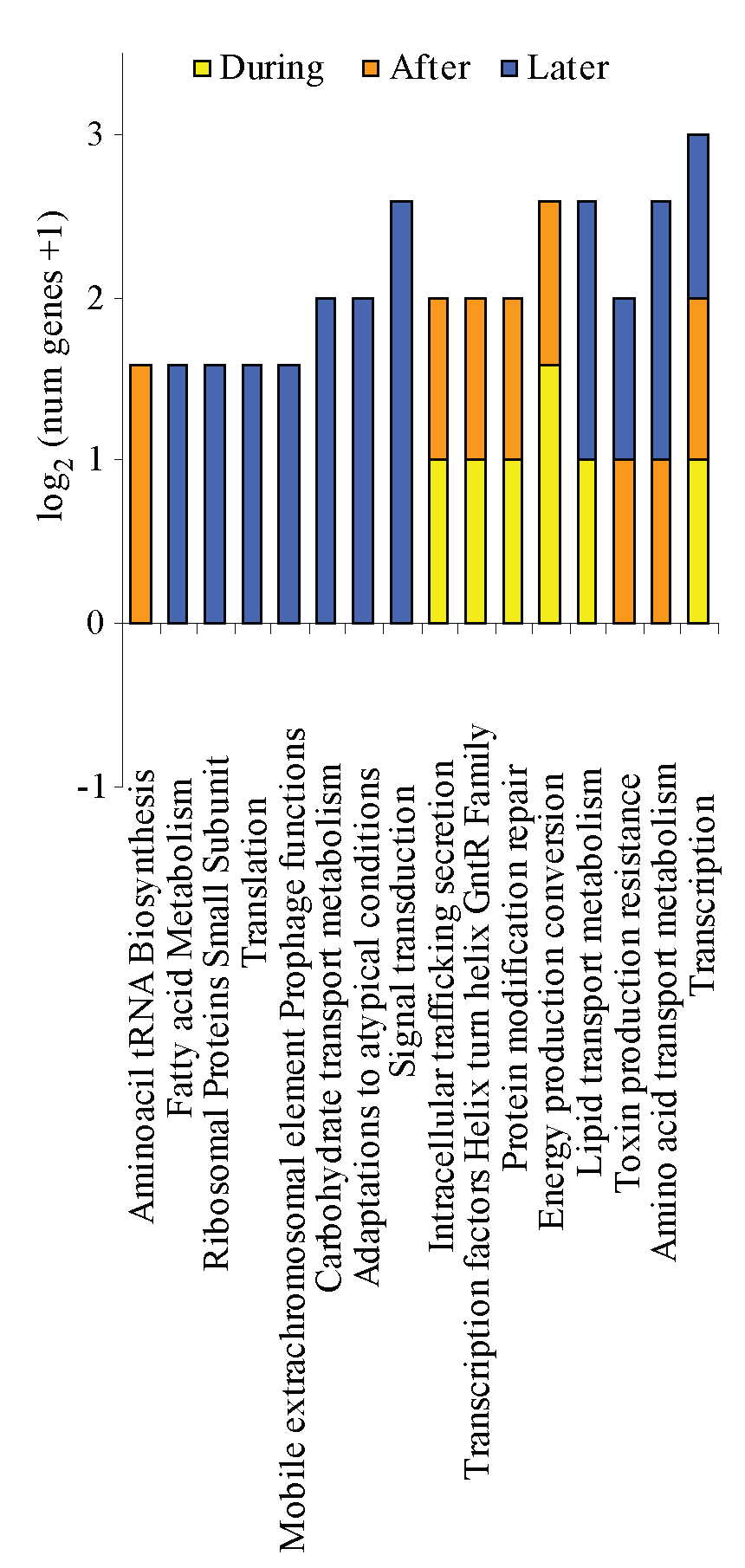

Supplement: Figure S1 — Metabolic pathways and cellular functions associated with those genes up-regulated during acid shock, immediately after acid shock ceased and 30 minutes after acid shock ceased. Columns had positive values if functions were up-regulated and negative if down-regulated. (TIF) [file pone.0051196.s001.tif]

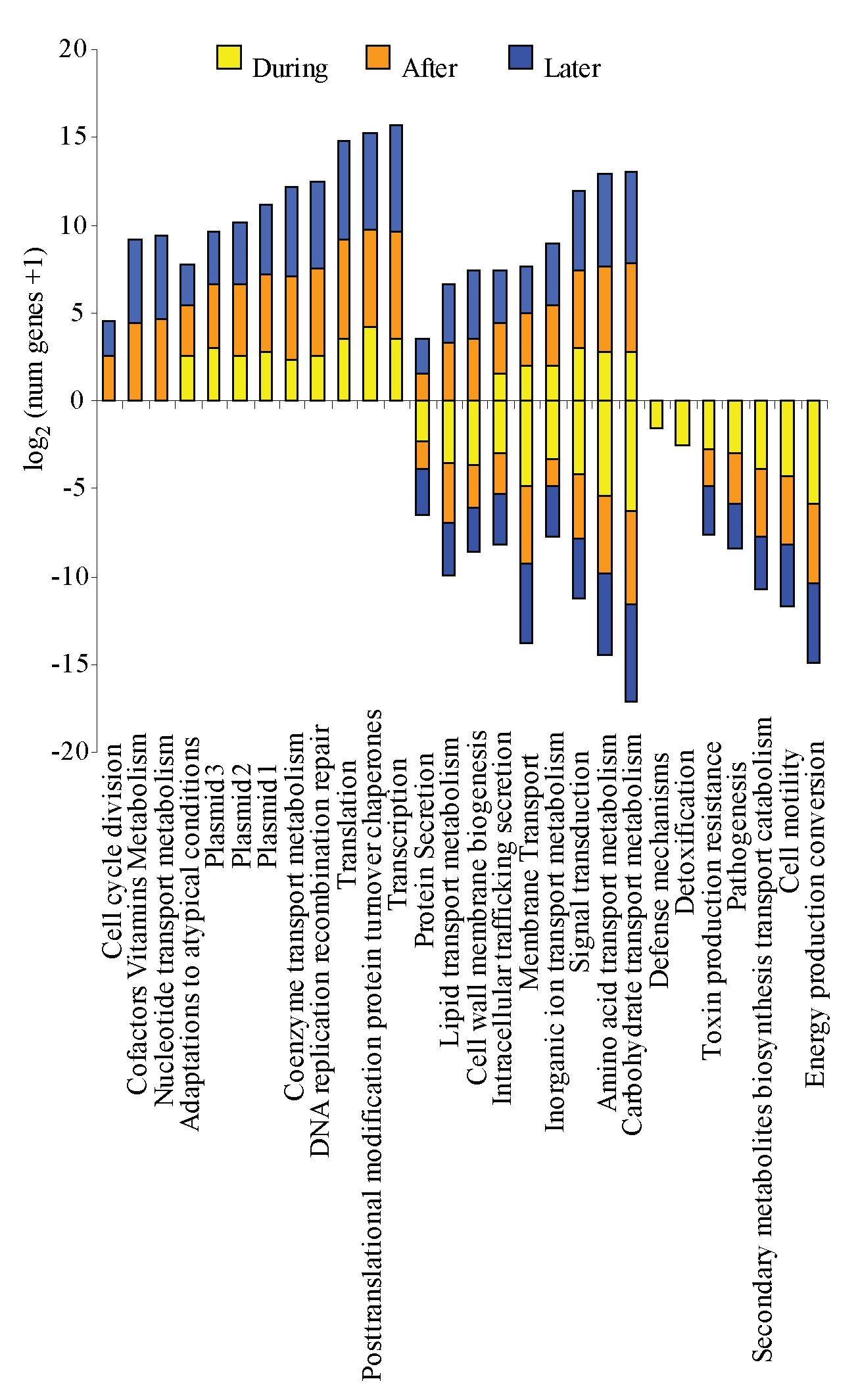

Supplement: Figure S2 — Main metabolic pathways and general cellular roles with a significant ( p <0.1) proportion of genes up- or down-regulated during heat stress (During), immediately (After) and 30 minutes (Later) after heat stress ceased. Columns had positive values if functions were up-regulated and negative if down-regulated. (TIF) [file pone.0051196.s002.tif]

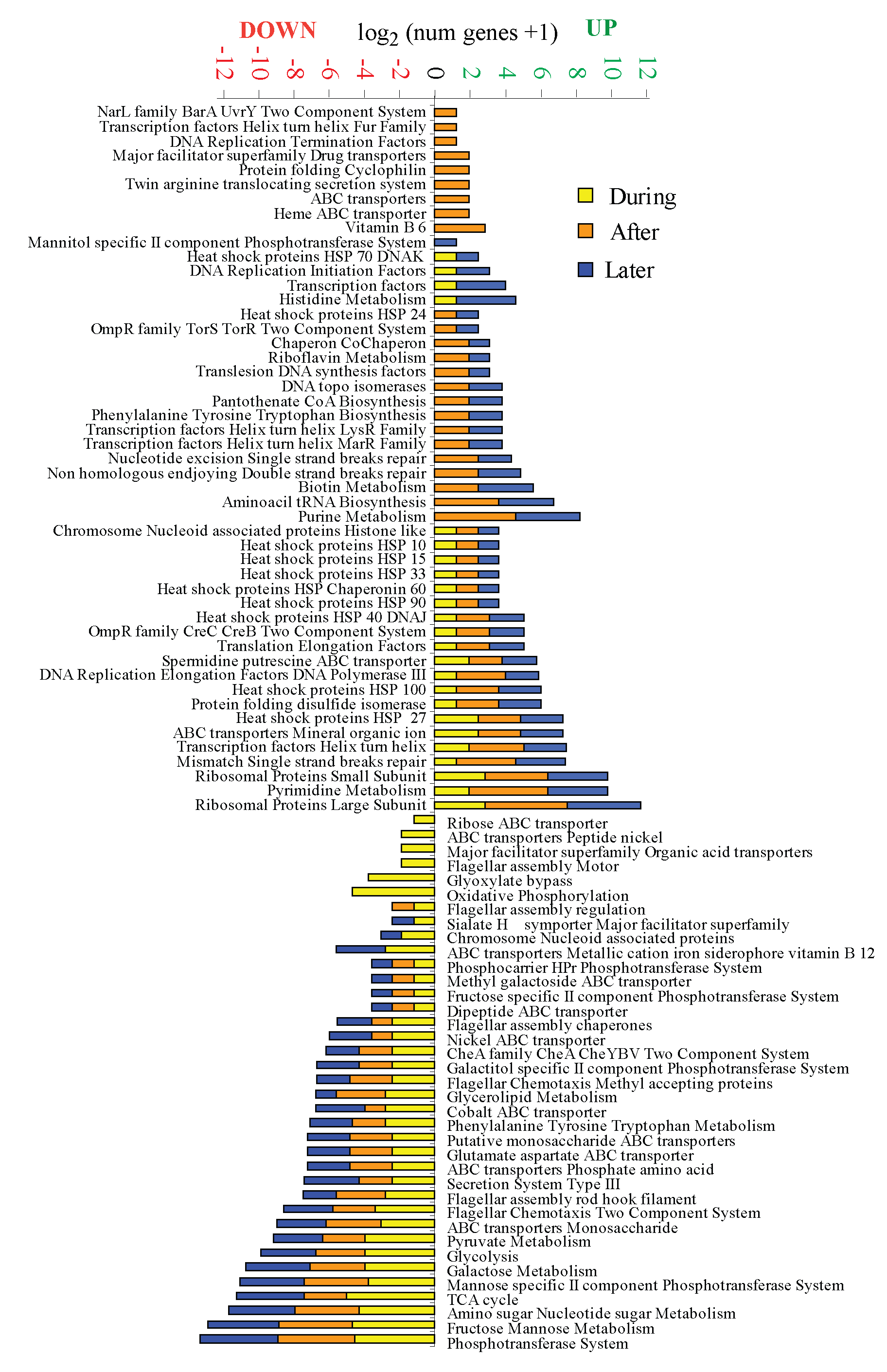

Supplement: Figure S3 — Specific metabolic pathways and cellular sub-roles with a significant ( p <0.1) proportion of genes up- or down-regulated during heat stress (During), immediately (After) and 30 minutes (Later) after heat stress ceased. Columns had positive values if functions were up-regulated and negative if down-regulated. (TIF) [file pone.0051196.s003.tif]

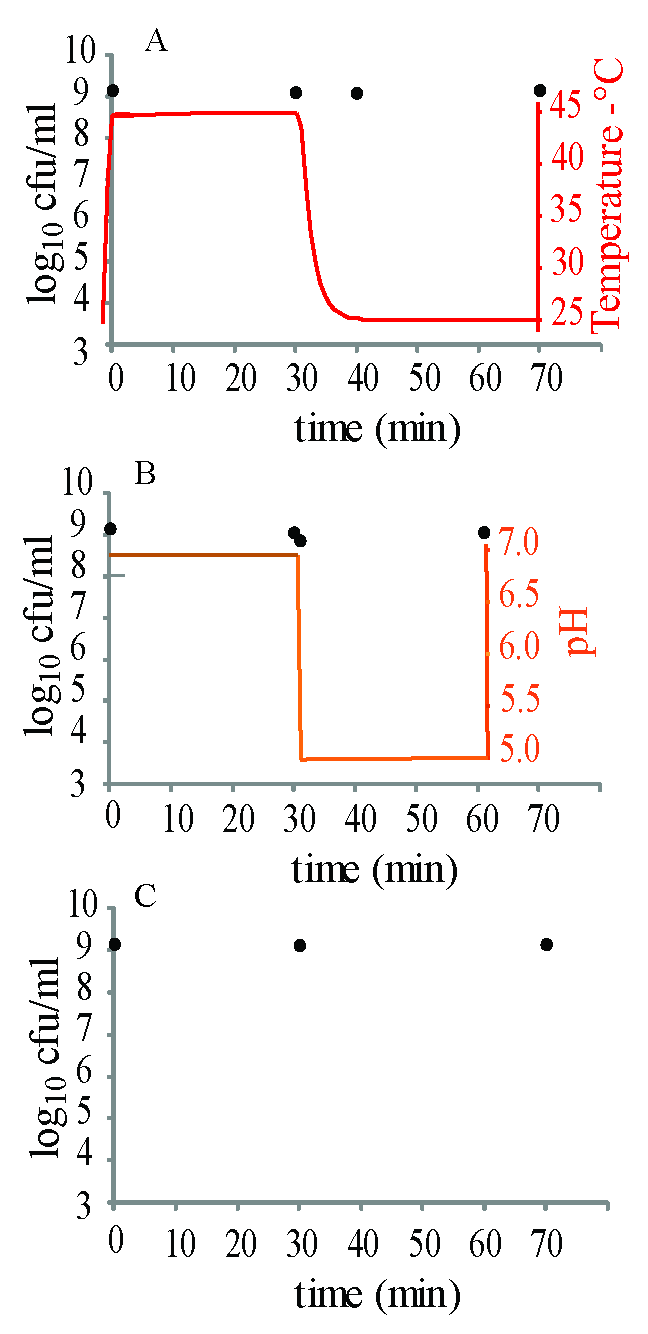

Supplement: Figure S4 — Bacterial concentration (closed symbols) during and after the exposure to stressing conditions: A) heat shock at 45°C 30 minutes and B) acidification of the medium at pH 5 for 30 minutes. Concentrations were also measured immediately after the cease of the stressing conditions and 30 minutes after resetting the original conditions as well as in untreated control populations maintained at 25 °C and pH 7 during the experimental course (C). Significant variation of the bacterial concentration was not detected in any population under any condition. (TIF) [file pone.0051196.s004.tif]
