# Supplementary material for: The Transcriptional Heat Shock Response of Salmonella Typhimurium Shows Hysteresis and Heated Cells Show Increased Resistance to Heat and Acid Stress
Source: PLoS One. 2012 Dec 7;7(12):e51196. doi: 10.1371/journal.pone.0051196 (PMC3517412; doi:10.1371/journal.pone.0051196)
Supplement: Table S1 — Number of replicated samples in which genes were up- or down- regulated because of heat stress (45°C). (PDF) [file pone.0051196.s005.pdf]

Table S1: Number of replicated samples in which genes were up- or down- regulated because of heat stress (45°C)

Gene name	Equivalent samples before stress (25°C)	30 min under stress (45°C)	Immediately after removing stress	30 min after removing stress
ackA		-3		
acnA		-3		-1
acnB		-3		
acrA			2	
add			2	1
adk	1		3	3
aer		-2	-2	-1
aes			2	1
agp		-3	-3	-1
agsA		3	3	3
ahpF			2	
aldB		-3	-3	-2
ansB		-3	-2	
araC			2	3
arcA			3	
aroH	1			2
aroK			3	
aroL			2	
asd			2	
asnS		1	3	3
aspA		-3	-1	
atpB			2	
avrA		2		
bioA	1		3	3
bioB			3	3
bioC				3
bioD				3
bioF			2	3
brnQ			2	1
cbiC		-3		
cbiF		-3	-2	-1
cbiG		-3		
cbiH		-3	-1	-1
cbiJ		-3	-1	-1
cbiK		-3	-2	-2
cbiL		-3	-1	-1
cbiM		-3	-2	-1
cbiN		-3	-1	-1
cbiO		-3		-1
cbiP		-2		
cboQ		-3		-2
cbpA		-2		
ccmB			2	1

ccmC			2	1
ccmE			2	1
cdd		1	3	3
celG	1	1	2	
cheB		-2	-1	-3
cheM		-3	-3	
cheR		-3	-2	
cheW		-2		-2
cheY		-3	-1	-3
cheZ		-2		-3
citG		1	2	2
clpB		3	3	3
clpP		1	3	3
clpX			3	2
cobS		-2		
creA		1	3	3
creB		1	3	3
creC			3	2
crp		-2	-1	
csdA			3	1
csiE		-3	-1	
cspA		2	3	1
cspC		-3		
cspE		-3		
csrA		-2		
cstA		-3	-1	
cydA		-3		
cydB		-3		
cyoA			2	1
cyoB			2	
cypD			3	
dapA		-3		
dapD		-3		
dcd			2	
dctA		-3	-2	-1
dcuA		-3		
deaD			3	3
def			3	
deoC			3	
dgoT		-3	-1	
dnaC			2	1
dnaG		1	3	3
dnaJ		3	3	3
dnaK		3	3	3
dnaX			2	
dppA		-3	-2	-3
dps		-3		-2
dxs			2	

ecnB		-1		-2
eco		-3		
eda			2	
emrA			3	2
emrD	1		2	
emrR			3	2
eutA		-1	-1	-3
eutB		-3	-2	-3
eutC		-3		-3
eutD		-3	-3	-3
eutE		-2	-2	-3
eutG		-1	-1	-3
eutH		-1		-3
eutJ		-3	-3	-3
eutK		-3	-2	-3
eutL		-3	-2	-3
eutM		-3	-3	-3
eutN		-3	-3	-3
eutP		-3	-3	-3
eutQ		-3	-3	-3
eutR		-1		-3
eutS		-1		-2
eutT		-3		-1
fabF			2	1
fba		-3		
fbp		-2		
fdhE		-3		
fdhF		-2		
fdoG		-3		
fdoH		-3		
fdoI		-3	-1	
fepE			2	1
fimA		-2	-3	-3
fkpA			2	
fldA			3	
fldB			2	1
flgK		-3	-2	
flgL		-3	-1	
flgM		-3	-2	
flgN		-3	-2	
fliC		-3	-3	-3
fliH			1	3
fliS		-1		-2
fliT		-1		-2
fljB		-3	-3	-3
flk			2	1
fmt			3	
fnr			3	

folD			2	
folE			3	
frdC		-2		
ftn			2	1
ftsI		-3	-1	
fumA		-3	-3	-1
fumC		-3	-2	-1
fur			3	1
fusA		1	3	3
fxsA			3	
galF			3	1
galK		-2		
galM		-3		-1
galP		-3	-2	-3
galT		-3		
gapA		1	3	2
gcvA			3	1
gcvH		-3		
gcvT		-2		
glgC		-3		-1
glgX		-3		
glmU			3	3
glnA			1	3
glnL				3
glnS			3	3
glpX				2
gltA		-3		
gltI		-3	-3	-3
gltJ		-3	-3	-2
gltK		-3	-3	-3
gltX			3	1
glyQ			2	
gmk			3	
gntU	1			2
gpt			3	1
greA			3	
greB			2	1
grpE			3	2
grxB		-3		
gsp			2	1
guaB			2	3
gutM			3	
gyrA			3	2
gyrB				2
hemA			2	
hemB			2	
hemL			3	
hemX		-3		

hepA		1	3	2
hflB			3	3
hha			2	
hilD		-3	-2	-3
hisB			1	3
hisC			1	3
hisD		1	3	3
hisG		1	3	3
hisH			1	3
hisI				2
hlpA		-3		
holB			2	
holC		1	3	3
holD			2	1
hrpA			2	1
hscB			3	3
hslU			3	3
hslV		1	3	3
htpG	1	3	3	3
htpX		2	3	3
htrB			2	1
hupA		-3		
hybA		-3	-1	
hybB		-3		
hybC		-3		
hybD		-3		
hybE		-3		
hybF		-3		
hycE				-2
hycH		-2		-2
hydN		-2		-2
hypA		-2		
hypB		-3		
hypC		-3		
hypD		-3		
hypO		-3		
iadA			1	3
iap			2	1
ibpA		3	3	3
ibpB		3	3	3
icdA		-3		
idnD		-1	-1	-2
idnO		-2	-3	-2
idnT		-1	-2	-1
ileS				3
ilvY			3	3
infA			2	1
infC		-3		

invB	-2		-3
invF	-3	-3	-3
invG			-2
invH	-3		-3
invI	-1		-3
invJ			-3
ispA		2	
katE	-3	-1	-1
katG	-3		
kdsB		3	1
kdtB		2	3
ldhA	2	3	3
leuS		2	
lipA	-2		
lipB		3	1
lon	1	3	3
lppB		2	1
lpxD	-3		
lrhA		3	3
lspA			3
lysA			2
maa		3	1
malE	-2		
marA	2	3	3
marB	1	3	3
marR	1	3	3
mdh	-3		
mdoG		3	
melA	-3	-3	-3
melB	-3	-3	-3
menA			2
mglB	-3	-3	-1
mlc		1	2
mopA	3	3	3
mopB	3	3	3
motA	-2		
motB	-2	-1	
mreB		3	
msrA	3	3	3
mtlA		1	3
mtlR			2
mutH		3	1
mutM	1	3	3
nagC		2	1
nagD		2	2
narP		3	1
nifS	1	3	3
nifU		3	3

nmpC	-3	-3	-3
nuoA	-3		
nuoB	-3		
nuoC	-3		
nuoE	-3		
nuoF	-3	-1	
nuoG	-3		
nuoH	-3		
nusA		1	3
ompC	-3	-2	-1
ompW	-3		
oppA	-3		
orf242		3	1
orgA	-2		-3
osmC	-3		-2
osmE	-3	-2	-3
osmY	-3	-1	-3
pckA	-3	-2	-1
pcnB		2	1
pdhR		3	
pduA	-2		
pduB	-3		
pduC	-3	-3	
pduD	-3	-3	
pduE	-3	-3	
pduG	-3	-3	
pduH	-2		
pduJ	-3	-2	-2
pduK	-3	-2	-2
pduL	-3	-2	-1
pduM	-3	-2	
pduN	-3	-1	
pduO	-3		
pduP	-3	-1	
pduQ	-3	-1	
pduS	-3	-1	
pduT	-3	-1	
pduU	-3	-1	
pduV	-3		
pduW	-3		-1
pdxB		2	1
pdxH		2	
pdxK	1	2	
pepD	-3		
pepT		2	1
pfkB	-2		
pflA		3	
pflB	-3		

pfs			2	1
pheS			3	2
phnR			2	1
phoH		-3		-1
phoL		1	3	3
phoP		-2		
phsB		-2		
pipC		-1	-1	-2
pldA			2	
pmgI				3
pncB				2
polB	1		2	
potC		2	3	2
potD		3	3	3
potE		-3	-3	-3
poxB		-2		-2
ppa			2	
pphB			2	
ppiA			3	1
prfA			3	1
prgI		-3	-3	-3
prgJ		-2	-1	-3
prgk		-3	-2	-3
prlC		2	3	3
proQ			3	1
proS			2	
prsA			2	
psiF		-3		-2
pspA		3	3	
pspB		3	3	
pspC		3	3	
pspD		3	3	
pta		-3		
purF	1		2	
purG			2	
purU			3	
pykF		-3		-1
pyrF	1		2	1
pyrG			3	1
rbsB		-3	-1	
rbsD				3
rcsF			3	1
recA			3	3
recF			3	3
rfaH			2	1
rhlB			2	
ribA			3	1
ribD			3	3

rimI	1		3	1
rnpA			3	
rob			2	
rplA			2	1
rplB		1	3	2
rplC			3	1
rplD		1	3	2
rplK			3	
rplM			2	1
rplN			3	1
rplT		-2		
rplU			3	1
rplW		1	3	1
rpmA			3	1
rpmB			2	
rpmE		3	3	3
rpmG			2	
rpmH			3	
rpoD		1	3	3
rpoE			2	
rpoH		3	3	2
rpoZ			2	
rpsA		2	3	1
rpsB		2	3	1
rpsF			2	
rpsG		2	3	3
rpsJ		1	3	2
rpsL		2	3	3
rpsT			2	1
rseA			3	
rseB			3	
rstA			2	
rsuA			3	1
rtcR			2	1
ruvA			2	1
sdaA			3	1
sdhA		-3		
sdhB		-3		
sdhC		-3		
sdhD		-3		
serC			2	
sicA		-1		-2
sipB		-3	-2	-3
sipC		-2	-2	-3
SL0322		-3	-2	
SL0351		-3		-3
SL0555			3	
SL0560		-3	-3	-3

SL0561	-3	-1	-3
SL0562	-3	-2	-2
SL0563	-3	-3	-3
SL0564	-3	-3	-3
SL0565	-3	-2	-2
SL0568		2	
SL0681	-3	-3	-3
SL0713	-3		
SL0794	-3		
SL0846		2	
SL0950		2	1
SL0983	-2		
SL1174		3	2
SL1235	-2		
SL1259	-3		
SL1391		3	
SL1399	-3	-2	
SL1475			2
SL1476	2	3	1
SL1478		3	1
SL1479	1	3	1
SL1480	1	3	
SL1537		3	1
SL1557		3	3
SL1558		3	
SL1596	2	1	
SL1603		3	3
SL1604	1	3	2
SL1605		3	3
SL1664		3	1
SL1721	1	1	2
SL1723	-3	-2	
SL1987		3	
SL1988		2	
SL2125		2	3
SL2157		3	1
SL2163	-3		
SL2283	-3	-2	
SL2330		2	3
SL2341	-3		-1
SL2409		3	1
SL2507		3	
SL2574	1	3	
SL2593		3	1
SL2594		2	
SL2631		3	
SL2632		2	
SL2633		3	

SL2674			-1	-3
SL2723		-3	-3	
SL2724		-3	-1	
SL2733			2	1
SL2763		2	3	
SL2781			2	
SL2784			3	
SL2850				-3
SL2884		2	3	2
SL2885		1	3	1
SL2919		-3	-2	
SL2921		-3	-2	
SL2966			2	
SL2998			2	1
SL3011			3	1
SL3012			3	1
SL3056				3
SL3057		1	3	3
SL3058		1	3	3
SL3059	1	3	3	3
SL3109			2	
SL3110			2	
SL3128		1	2	
SL3143		3	3	3
SL3144				3
SL3149	1		2	1
SL3219	1			2
SL3226		-3	-3	-3
SL3227		-3	-3	-3
SL3228		-3	-3	-3
SL3229		-3	-3	-3
SL3230		-3	-3	-3
SL3231		-3	-3	-3
SL3232		-3		-3
SL3233		-2	-1	-3
SL3234				-3
SL3334	1	1	3	2
SL3378		1	3	3
SL3524		2	3	
SL3566		-3		
SL3617			3	
SL3618			3	
SL3743	1	3	2	
SL3744		2	2	
SL3750				2
SL3786		1	2	
SL3808	1		3	
SL3867		1	3	

SL3976		3	3	
SL3977		3	1	
SL3979		1	2	
SL3985		-2		
SL3993		-3	-3	-1
SL4014	1	2	3	3
SL4015		1	3	3
SL4016			1	3
SL4020		-3	-3	-3
SL4029		-3	-3	-3
SL4222		-2		
SL4253		1	2	
SL4395		-3	-2	
SL4396		-3	-2	
SL4397		-3	-1	
SL4421			3	1
SL4422			2	
SL4426			2	1
SL4441		1	3	3
SL4460		2	3	
SL4466		-3	-3	
SL4467		-3	-3	-1
SL4468		-3	-3	-1
SL4469		-3	-3	-3
SL4470		-3	-3	-3
SL4471		-3	-3	-3
SLP1_0008			2	
SLP1_0009		2	2	
SLP1_0054			3	3
SLP1_0055			3	3
SLP1_0056		1	2	
SLP1_0057		2	3	3
SLP1_0058		3	3	3
SLP1_0059		3	3	2
SLP1_0060		1	3	3
SLP1_0062			3	
SLP1_0063		1	3	
SLP1_0072			3	
SLP1_0074				2
SLP1_0075		1	3	3
SLP1_0076			3	3
SLP1_0077		1	3	3
SLP1_0079		3	3	3
SLP1_0080		3	3	3
SLP1_0083			2	
SLP1_0091			3	
SLP1_0092			3	2
SLP1_009[illegible]	1		2	2

SLP1_009	1			2
SLP2_0003		1	3	
SLP2_0004		3	3	
SLP2_0005			3	
SLP2_0010			2	
SLP2_0016			3	3
SLP2_001	1	1	3	3
SLP2_0019		1	2	
SLP2_0020		3	3	1
SLP2_0021		3	3	3
SLP2_0022		3	3	3
SLP2_0023			3	2
SLP2_0024			2	1
SLP2_002	1			2
SLP2_0048			3	2
SLP2_0049			3	2
SLP2_0055		3	3	
SLP2_007	1			2
SLP2_0095				2
SLP2_0098				2
SLP3_0001			2	
SLP3_0003		1	3	3
SLP3_0004		2	3	3
SLP3_0005		2	3	3
SLP3_000	1	3	3	3
SLP3_0007		3	3	3
SLP3_000	1	3	3	3
SLP3_000	1	3	3	3
SLP3_0011		3	3	1
SLP3_001	1		2	1
SLP3_001	1		2	1
slpA				2
smpA			2	
smpB			2	
sodB		-3		
sopB				-3
soxR			3	2
spaO		-1		-3
spaP				-2
speF		-3	-3	-3
speG			2	
sptP		1	2	
spy		1	3	
srlR			2	
sseE			2	
stpA		3	3	3
sucA		-3		-1
sucB		-3	-1	-1

sucC		-3		-1
sucD		-3		-1
suhB	1		2	1
tatA			2	1
tatB			2	
tbpA		1	2	
tcp		-3	-3	-3
tesB			2	1
thiL	1		2	
thrS		-3		
tktB		-2		-1
tmk			3	
tolC		-3		
tolQ	1		3	1
topA	1		2	
torR			3	3
tpx		-2		
treA		-3	-1	-1
treR			2	
trxA			2	1
trxB			3	
trxC			2	1
tsf			3	1
tus		1	3	1
typA			3	1
ubiC			2	
ubiG			2	
ucpA		-3	-2	
udk			3	1
uvrA			1	3
uvrC	1		3	2
uvrY			2	1
uxuR			2	
valS				3
wcaI				2
wraB				-2
xseA			3	3
yaaA			2	1
yacC			3	1
yacE	1		3	1
yacF			2	
yadF	1		2	1
yadR			3	1
yaeB			3	
yaeH		-3		-1
yaeQ			2	1
yafA			2	
yafD			3	1

yafK	1		3	1
yahO		-2		
yaiI			3	
yajO		-3	-2	-1
ybaD			3	2
ybaJ			2	
ybaM			2	
ybaO			3	1
ybaY		-2	-2	-3
ybbB			2	
ybbM	1		3	2
ybbN		3	3	3
ybbO			2	
ybdD		-3		
ybdF			2	
ybdG			2	1
ybdN		1	2	
ybeA			3	1
ybeB			3	
ybeD		1	3	3
ybeL		-2		
ybeX			3	3
ybeY			3	3
ybfA		1	3	2
ybgE		-3		
ybgS		-1		-2
ybgT		-2		
ybhQ		-3		
ybiJ		1	3	3
ybiS			2	
ybiV(1)			3	
ycaR			3	1
yccA			2	1
yccD		-3		
yccJ				-2
yccS			2	1
yccV		1	3	3
yceG			3	
yceP			2	1
ycfD			2	
ycfF		-2		
ycfL		-2		
ycfQ			3	
ycfR	1	3	3	3
ycgB		-3		-1
ychF			3	1
yciM			3	1
yciS			3	1

yciT	1		2	
ycjF			3	3
ycjX		2	3	3
ydaL			3	1
ydbH		1	3	1
ydcX			3	
ydeA	1		3	1
ydeV		-3	-3	-1
ydeW		-3	-2	
ydeY		-3	-2	-1
ydeZ		-3	-3	-3
ydfH		-2		
ydfZ		3	3	1
ydgA		-3		
ydgM			2	
ydgO				2
ydhC			2	
ydiH			3	2
ydiZ		-2		
yeaD			3	3
yeaG		-3	-2	-1
yeaH		-3	-1	-1
yeaM			2	
yeaO		1	2	1
yebK			1	2
yecG		-3		
yecH			1	2
yedJ		1	3	1
yeeI		-3		
yegH			3	2
yegQ			3	2
yegT			1	3
yegU			1	3
yehE		2	3	
yehS			2	1
yeiA		-3		
yeiH			3	2
yejG			2	
yejH			2	
yejL			2	
yejM			3	
yfbQ			2	
yfcE			3	
yfcL		2	3	1
yfcX			3	2
yfcZ		-3	-2	
yfeC		-3		
yfeD		-2		

yfgB			2	
yfhB		-2		
yfhF			3	3
yfhP		1	3	2
yfiO			3	
yfjF			2	
yfjG			2	1
ygaA		2	3	3
ygaC	1		3	1
ygaD		1	3	3
ygaU		-3		-2
ygbI			2	1
ygcF	1		3	1
ygcH		-3		
ygdD			3	
ygdE			3	1
ygdH		-3		
ygdI		-3	-2	-3
ygfB			3	
ygfE		-3		
yghA		-3	-3	-2
yghJ		-2	-2	-1
ygiC		-3		
yhbC			3	2
yhbS		-2		
yhbU			2	
yhcN		1	3	2
yhcO		-3		
yhdN		1	3	3
yheL		2	3	3
yheT			3	2
yheU			2	1
yhgI		2	3	3
yhhV		3	3	1
yhiI		-3		
yhiQ			3	3
yhiR			2	
yhjC	1		2	1
yhjH		-2		
yhjT			3	
yiaG		-3		-2
yidA			3	3
yigC			2	
yihD		-2		
yiiL		-3		
yjbO		2	1	
yjcB		2	3	1
yjfN		-3	-2	

yjfO	-3	-2	
yjiE		2	3
yjiG		3	
yjiH		3	
yjjY		2	
ylaC		2	
ymfC		3	1
ynaF	-3	-2	-3
yneA	-3	-3	-3
yneB	-3	-3	-3
yneC	-3	-3	-3
ynfK		3	1
yobF	-3		
yohJ		3	1
yohK	1	3	2
yqfA	-3		
yqiA		2	1
yqiB		2	
yqiE	1	3	1
yqjC	-3		
yqjK	-3		-1
yrbL			2
yrdC	-3		
yrfH	1	3	3
yrfI	1	3	3
ytfH	1	3	1
ytfL		3	2
zntR	3	3	3
znuC		3	1
