# Supplementary material for: The Transcriptional Heat Shock Response of Salmonella Typhimurium Shows Hysteresis and Heated Cells Show Increased Resistance to Heat and Acid Stress
Source: PLoS One. 2012 Dec 7;7(12):e51196. doi: 10.1371/journal.pone.0051196 (PMC3517412; doi:10.1371/journal.pone.0051196)
Supplement: Table S2 — Number of replicated samples in which genes were up- or down- regulated because of acid stress (pH 5) and comparison with results of expression under heat stress in Table S1. (PDF) [file pone.0051196.s006.pdf]

Table S2: Number of replicated samples in which genes were up- or down- regulated because of acid stress (pH 5) and comparison with results of expression under heat stress in Supplementary Table S1

Gene name	Equivalent samples before stress (pH 7)	30 min under stress (pH 5)	Immediately after removing acid stress	30 min after removing acid stress	30 min under stress (45°C)	Immediately after removing heat stress	30 min after removing heat stress
aceA				2			
ahpC		1	2				
exbB		2	3				
fadB				2			
feoA		1	2				
hycA		1	2				
hydN		3	2		-2		-2
hypA		2	3		-2		
ibpB				2	3	3	3
mglB		-1	-2		-3	-3	-1
pdhR		3	3			3	
pduA		2	1		-2		
pduD		2			-3	-3	
pspA				3	3	3	
pspB				3	3	3	
pspC				3	3	3	
pspD				3	3	3	
pstS				2			
putA				2			
ribB				2			
rpsB				2	2	3	1
rpsP				2			
rseA				3		3	
rseB				2		3	

SL0452		2					
SL4222	1	2			-2		
SLP3_0012		2					
sprB			2				
yafH			2				
ybaJ	1	2				2	
ycbK			2				
ycfR	3	3			3	3	3
ydiH		2				3	2
yehE		2			2	3	
yeiH	3	3				3	2
yfbE			2				
ygiM			2				
yohJ	2	3				3	1
yrdC	2				-3		
